# Persistence of Covert but Not Overt Corrective Saccades in Darkness: Insights From Unilateral and Bilateral Vestibular Loss

**DOI:** 10.7759/cureus.112860

**Published:** 2026-07-17

**Authors:** Francisco Zuma e Maia, Bernardo F Ramos

**Affiliations:** 1 Otolaryngology, Clinica Maia, Canoas, BRA; 2 Otolaryngology, Federal University of Espirito Santo, Vitoria, BRA

**Keywords:** bilateral vestibular loss, cervico-ocular reflex, covert saccades, overt saccades, unilateral vestibular loss, vestibulo-ocular reflex, vhit, video head impulse test

## Abstract

Corrective saccades identified during the video head impulse test (vHIT) are commonly classified as overt or covert according to their timing relative to head movement. Although overt saccades are generally considered visually driven, the mechanisms underlying covert saccades remain incompletely understood. We report two illustrative cases of vestibular hypofunction evaluated with vHIT under standard illuminated conditions and in complete darkness. The first patient had unilateral vestibular loss, whereas the second had bilateral vestibular loss. In both cases, overt corrective saccades observed during testing in light conditions disappeared in darkness, while covert saccades remained detectable. These findings are consistent with the hypothesis that overt and covert corrective saccades may rely, at least in part, on different physiological mechanisms. The abolition of overt saccades following elimination of visual feedback is consistent with a visually mediated origin. In contrast, the persistence of covert saccades suggests participation of non-visual pathways involved in gaze stabilization. The observed latencies were compatible with previously reported latencies of cervical proprioceptive responses, including the cervico-ocular reflex (COR), although a direct causal relationship cannot be established from the present observations. This simple light-versus-darkness vHIT protocol may provide a clinically accessible approach for exploring compensatory gaze stabilization mechanisms in patients with vestibular disorders.

## Introduction

The vestibulo-ocular reflex (VOR) is the primary mechanism responsible for maintaining visual stability during rapid head movements, generating compensatory eye movements with latencies of approximately 10-15 ms. The video head impulse test (vHIT), introduced as a quantitative extension of the bedside head impulse test described by Halmagyi and Curthoys, allows objective assessment of semicircular canal function through high-speed video-oculography and provides simultaneous measurement of VOR gain and corrective saccades [1,2].

In patients with peripheral vestibular hypofunction, corrective saccades are commonly classified as overt or covert according to their timing relative to the head impulse. Overt saccades occur after completion of the head movement and are often visible during bedside examination. In contrast, covert saccades occur during the head impulse itself and generally require high-speed recording for detection [3,4]. Since their initial description, covert saccades have attracted considerable interest because they appear to represent an adaptive compensatory mechanism that contributes to gaze stabilization despite reduced vestibular function.

Although overt saccades are generally believed to depend on visual error detection following retinal slip, the neural mechanisms responsible for covert saccade generation remain incompletely understood. Several non-visual mechanisms have been proposed, including predictive motor strategies based on efference-copy signals, cervical proprioceptive inputs, and adaptive central nervous system processes [5-7]. Among these, the cervico-ocular reflex (COR) has received particular attention because its latency falls within a temporal range compatible with the occurrence of covert corrective eye movements. Previous studies have demonstrated enhancement of the COR in patients with vestibular hypofunction, suggesting a compensatory role when vestibular inputs are reduced [8,9].

If overt corrective saccades are primarily dependent on visual feedback, whereas covert corrective saccades rely predominantly on non-visual mechanisms, elimination of visual input should selectively affect overt saccades while preserving covert responses. Despite increasing recognition of covert corrective saccades, the relative contribution of visual and non-visual mechanisms to their generation remains incompletely understood. Therefore, comparing vHIT recordings obtained under illuminated and complete darkness conditions may provide clinically relevant insight into whether visual feedback is necessary for the generation of different types of corrective saccades. To our knowledge, few reports have directly explored this phenomenon using routine clinical vHIT equipment under both illuminated and complete darkness conditions.

We present two illustrative cases, one involving unilateral vestibular loss and the other bilateral vestibular loss, in which vHIT recordings were obtained under light and dark conditions. The observations provide clinical evidence supporting a dissociation between overt and covert corrective saccades and offer additional insights into the potential contribution of non-visual mechanisms to compensatory gaze stabilization.

## Case presentation

Two adult patients with documented peripheral vestibular hypofunction were evaluated at a tertiary dizziness clinic using the vHIT. Testing was performed with the Natus ICS Impulse system (Natus Medical Inc., Pleasanton, CA, USA), which utilizes high-speed infrared video-oculography at approximately 250 frames per second. The system simultaneously records head and eye velocity, calculates VOR gain, and identifies corrective saccades and their respective latencies.

For both patients, vHIT was performed under two sequential conditions. In the illuminated condition (LIGHT), patients were instructed to fixate on a stationary target positioned approximately 1.5 meters ahead in the mid-sagittal plane. In the darkness condition (DARK), all visible light sources were eliminated, and patients were instructed to maintain gaze in the direction of the previously viewed target. The LIGHT condition was always performed first, followed by the DARK condition. Approximately 15-20 unpredictable horizontal head impulses were delivered to each side at peak velocities ranging from 150°/s to 250°/s.

Corrective saccades were classified according to their temporal relationship with the head impulse, following conventional vHIT definitions. Saccades occurring before completion of the head impulse were classified as covert, whereas those occurring after completion of the head impulse were classified as overt. Classification was based on visual inspection of the synchronized head and eye velocity traces generated by the ICS Impulse software. All recordings were reviewed by the same experienced examiner using identical classification criteria under both testing conditions.

Case 1

The patient was a 64-year-old woman who presented with acute vestibular syndrome and was evaluated three days after symptom onset. vHIT findings were consistent with acute left unilateral vestibular hypofunction confirmed by caloric testing demonstrating significant unilateral canal paresis, with vestibular neuritis considered the most likely etiology. There was no history of central neurological disease, cervical spine pathology, or oculomotor disorders. Following diagnosis, she was promptly referred for vestibular rehabilitation therapy. A follow-up vHIT performed three months later demonstrated no substantial recovery of VOR gain in the affected left lateral semicircular canal.

**Figure 1:**
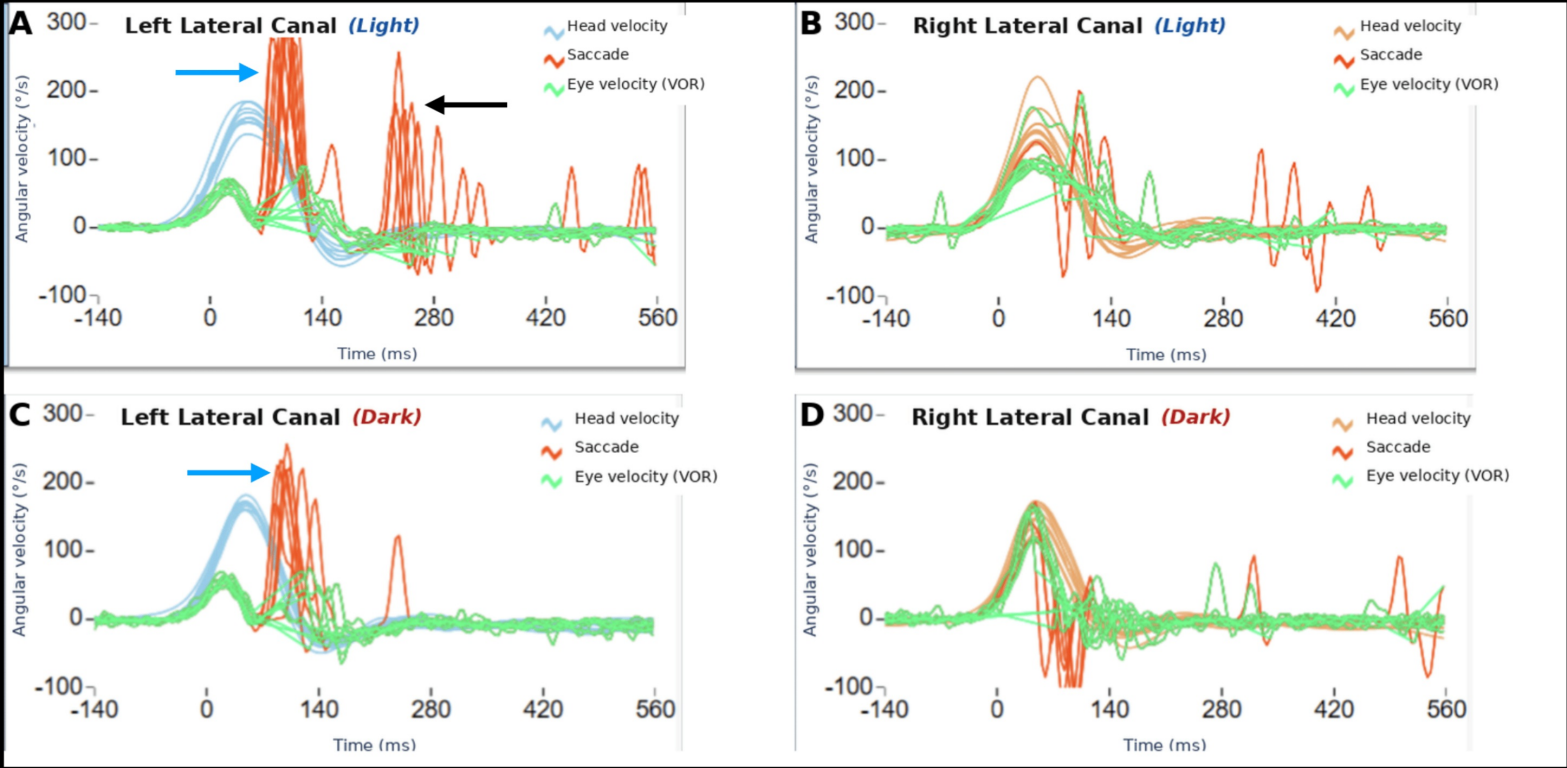
vHIT of Case 1 recordings obtained from a 64-year-old woman with left acute unilateral vestibular hypofunction. A: Left lateral semicircular canal tested under illuminated conditions. Mean VOR gain was 0.36, with covert saccades exhibiting a mean latency of 104 ms. B: Right lateral semicircular canal tested under illuminated conditions. Mean VOR gain was 0.79, without corrective saccades. C: Left lateral semicircular canal tested in complete darkness. Mean VOR gain was 0.24, with covert saccades exhibiting a mean latency of 92 ms. D: Right lateral semicircular canal tested in complete darkness. Mean VOR gain was 0.31, without corrective saccades. The x-axis represents time (ms), and the y-axis represents angular velocity (°/s). Gain represents the ratio between eye velocity and head velocity. Corrective saccade latency indicates the time interval between the onset of the head impulse and the onset of the corrective eye movement. The blue arrow indicates covert saccades and the black arrow indicates overt saccades. vHIT, video head impulse test; VOR, vestibulo-ocular reflex.

Under illuminated conditions (Figure 1), the affected left side demonstrated a markedly reduced VOR gain of 0.36 accompanied by prominent corrective saccades with a mean latency of 104 ms. Both early and late corrective eye movements were observed, consistent with covert and overt saccadic components. The contralateral side demonstrated a VOR gain of 0.79 with fewer corrective saccades.

When testing was repeated in complete darkness (Figure 1), a clear dissociation between corrective saccade types became apparent. On the affected side, VOR gain decreased further to 0.24, and overt corrective saccades were no longer observed. However, early corrective saccades remained present, with a mean latency of 92 ms. On the contralateral side, VOR gain decreased from 0.79 to 0.31, while early corrective saccades continued to be detectable.

The findings in this patient demonstrated three consistent observations: VOR gain was lower in darkness than in light; overt corrective saccades disappeared when visual feedback was removed; and covert corrective saccades persisted despite elimination of visual input.

Case 2

The patient was a 40-year-old man who presented with chronic imbalance and oscillopsia four months after the onset of bilateral vestibular dysfunction secondary to gentamicin ototoxicity. Gentamicin had been administered during treatment for a bacterial intra-abdominal infection. vHIT findings were consistent with bilateral vestibular hypofunction, confirmed by absent caloric responses bilaterally. No history of cervical disease, central neurological disorder, or ocular motor abnormalities was present. Following diagnosis, he was promptly referred for vestibular rehabilitation therapy. A follow-up vHIT performed three months later demonstrated no substantial recovery of VOR gain in either lateral semicircular canal.

**Figure 2:**
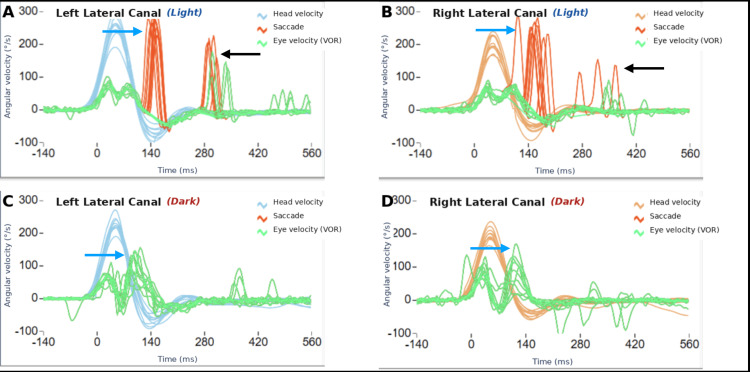
vHIT of Case 2 recordings obtained from a 40-year-old man with bilateral vestibular hypofunction. A: Left lateral semicircular canal tested under illuminated conditions. Mean VOR gain was 0.34, with covert saccades exhibiting a mean latency of 156 ms. B: Right lateral semicircular canal tested under illuminated conditions. Mean VOR gain was 0.38, with convert saccades exhibiting a mean latency of 112 ms. C: Left lateral semicircular canal tested in complete darkness. Mean VOR gain was 0.32, with covert saccades exhibiting a mean latency of 104 ms. D: Right lateral semicircular canal tested in complete darkness. Mean VOR gain was 0.36, with covert saccades exhibiting a mean latency of 92 ms. The x-axis represents time (ms), and the y-axis represents angular velocity (°/s). Gain represents the ratio between eye velocity and head velocity. Corrective saccade latency indicates the time interval between the onset of the head impulse and the onset of the corrective eye movement. The blue arrow indicates covert saccades and the black arrow indicates overt saccades. vHIT, video head impulse test; VOR, vestibulo-ocular reflex.

In the illuminated condition (Figure 2), vHIT demonstrated bilateral corrective saccades superimposed on deficient VOR responses. Corrective saccade latencies were approximately 156 ms on the left side and 112 ms on the right side.

When testing was repeated in complete darkness (Figure 2), overt corrective saccades were no longer evident on either side. In contrast, early corrective eye movements occurring within the head impulse interval remained detectable bilaterally. Although visual feedback had been eliminated, covert corrective responses persisted despite severe bilateral vestibular impairment.

The persistence of covert saccades in this patient was particularly noteworthy because compensatory eye movements remained detectable even in the setting of profound vestibular dysfunction and absence of visual fixation. The pattern observed was similar to that identified in the patient with unilateral vestibular loss, suggesting that covert and overt corrective saccades may rely on different physiological mechanisms.

## Discussion

The principal finding of this report was the consistent dissociation between overt and covert corrective saccades observed under illuminated and complete darkness conditions. In both patients, elimination of visual feedback resulted in the disappearance of overt corrective saccades, whereas covert corrective saccades remained detectable. This pattern was observed in both unilateral and bilateral vestibular loss, suggesting that the two types of corrective eye movements may depend, at least in part, on different physiological mechanisms [3,4]. As a descriptive case report involving two patients, the present observations should be regarded as hypothesis-generating and interpreted with appropriate caution.

Overt corrective saccades are generally considered a response to retinal slip occurring after completion of the head impulse. When vestibular function is impaired, gaze fails to remain aligned with the visual target during head rotation, generating a visual error signal (retinal slip) that subsequently triggers a corrective saccade. Because this mechanism depends on visual information, elimination of visual feedback would be expected to reduce or abolish overt corrective responses. In healthy individuals, vHIT typically demonstrates normal VOR gain values, and both overt and covert corrective saccades are generally absent during properly performed head impulses. In contrast, both patients in the present report exhibited reduced VOR gain together with corrective saccades, with the persistence of covert saccades in darkness representing the principal observation of this study. The findings observed in both patients are consistent with this interpretation and with current models of visually driven saccadic correction [7].

In contrast, covert saccades persisted despite the absence of visual input. Since these corrective eye movements occurred during the head impulse itself, their generation appears unlikely to depend exclusively on visual error processing. Several non-visual mechanisms have been proposed to explain covert saccade generation, including predictive motor control strategies, efference-copy signals, cervical proprioceptive input, and adaptive cerebellar processes [5,6,8,10,11]. The present observations cannot distinguish among these mechanisms; however, they provide additional support for the concept that covert saccades are at least partially independent of visual feedback.

Previous studies have reported covert corrective saccade latencies typically ranging from approximately 70 to 120 ms, whereas overt corrective saccades usually occur between 150 and 250 ms [3,4,8]. The latencies observed in the present cases fall within these previously reported ranges, further supporting the physiological plausibility of the observed response patterns.

One particularly interesting candidate mechanism is the COR. The COR produces compensatory eye movements in response to cervical proprioceptive stimulation and has been shown to become enhanced in patients with vestibular hypofunction [12,13]. Previous studies have demonstrated that the contribution of the COR is normally small in healthy individuals but may increase substantially following vestibular loss as part of the compensatory process [5,6,12,13]. The latencies observed for covert saccades in the present report were compatible with previously reported COR response latencies, supporting the possibility that cervical proprioceptive pathways contribute to these early compensatory eye movements [5,6].

The findings observed in the patient with bilateral vestibular loss deserve particular consideration. In this patient, covert saccades remained detectable despite severe vestibular dysfunction and elimination of visual feedback. This observation is consistent with the hypothesis that alternative non-visual mechanisms may contribute to gaze stabilization when vestibular input is markedly reduced [5,6,12,13]. Although the present study cannot directly establish the relative contribution of cervical proprioception, efference-copy mechanisms, or cerebellar predictive processes, the persistence of covert responses under these conditions supports further investigation of these pathways [10,11].

An additional consideration concerns the anatomical organization of the principal vestibular and cervical reflexes involved in head and gaze stabilization. The VOR and the COR share a common characteristic: both ultimately project to the oculomotor system and are capable of generating compensatory eye movements. In contrast, the vestibulo-collic reflex (VCR) and cervico-collic reflex (CCR) primarily target cervical musculature and contribute to stabilization of head position rather than gaze [14].

The VCR is driven by vestibular input originating from the semicircular canals and otolithic organs, but its efferent projections are directed predominantly to cervical motor pathways through the vestibulospinal system. Similarly, the CCR is driven by cervical proprioceptive input and contributes primarily to stabilization of head posture through activation of cervical musculature. In contrast, the COR utilizes cervical proprioceptive information to generate compensatory eye movements through projections involving vestibular nuclei and oculomotor pathways [14].

This anatomical distinction may be relevant when considering potential mechanisms underlying covert corrective saccades. Because covert saccades represent compensatory eye movements, reflex pathways possessing direct oculomotor outputs are physiologically more plausible contributors than pathways whose primary output is directed to neck musculature. Although these anatomical considerations do not establish the COR as the exclusive source of covert saccades, they support the hypothesis that cervical proprioceptive pathways may participate in the generation of early compensatory eye movements, particularly under conditions in which visual feedback is absent and vestibular function is impaired [12-14].

An additional observation was the systematic reduction in measured VOR gain during testing in darkness. Although the study was not designed specifically to investigate this phenomenon, the findings suggest that visual fixation may contribute to gaze stabilization during standard illuminated testing. If confirmed in larger studies, dark-condition vHIT could potentially provide complementary information regarding the severity of vestibular impairment by minimizing visual contributions to eye stabilization [1,4].

From a clinical perspective, the light-versus-darkness protocol described here is simple, inexpensive, and easily reproducible using commercially available vHIT systems [1,4]. Beyond its potential research applications, this approach may provide additional insight into compensatory strategies used by individual patients with vestibular dysfunction and could help guide future rehabilitation strategies targeting non-visual gaze stabilization mechanisms [15].

Several limitations should be acknowledged. First, this report describes only two patients and should therefore be interpreted as a descriptive clinical observation rather than a hypothesis-testing study. Consequently, the findings cannot establish causality or be generalized to the broader population of patients with vestibular disorders.

Although the persistence of covert saccades in darkness is consistent with the involvement of non-visual compensatory mechanisms, the present report was not designed to directly identify the neural substrates responsible for covert saccade generation. Cervical proprioceptive activity, cerebellar function, and efference-copy mechanisms were not measured directly. Therefore, the proposed contribution of the COR should be considered a physiologically plausible interpretation rather than a definitive demonstration.

Future studies involving larger cohorts, healthy controls, and dedicated neurophysiological methodologies will be necessary to clarify the specific mechanisms underlying covert corrective saccades and to determine the relative contribution of cervical proprioception, predictive motor control, and vestibular compensation processes.

## Conclusions

This report describes a consistent pattern of corrective saccade behavior during vHIT evaluation under illuminated and complete darkness conditions. In both unilateral and bilateral vestibular loss, overt corrective saccades disappeared following elimination of visual feedback, whereas covert corrective saccades remained detectable.

Unlike healthy individuals, who typically exhibit normal VOR gain values without corrective saccades during properly performed vHIT, both patients demonstrated reduced VOR gain together with persistent covert corrective saccades in darkness despite different clinical etiologies. These observations are consistent with the hypothesis that overt and covert corrective saccades may rely, at least in part, on different physiological mechanisms.

The observed findings are compatible with a potential contribution of non-visual compensatory pathways, including the COR, to covert corrective saccade generation; however, the present descriptive case report cannot establish a direct causal relationship or identify the specific mechanisms involved. These observations should therefore be regarded as hypothesis-generating and warrant further investigation in larger controlled studies.
